# Supplementary material for: Polymeric Carriers Designed for Encapsulation of Essential Oils with Biological Activity
Source: Pharmaceutics. 2021 Apr 28;13(5):631. doi: 10.3390/pharmaceutics13050631 (PMC8146382; doi:10.3390/pharmaceutics13050631)
Supplement: Supplementary file 1 [file pharmaceutics-13-00631-s001.zip › pharmaceutics-1194557-supplementary.pdf]

pharmaceutics

MDPI

Supplementary Materials: Polymeric Carriers Designed for Encapsulation of Essential Oils with Biological Activity

Aurica P. Chiriac, Alina G. Rusu, Loredana E. Nita, Vlad M. Chiriac, Iordana Neamtu and Alina Sandu

Table 1. The table presenting the polymeric matrices utilized for essential oils (EOs) encapsulation was made based on the documentation performed for the realization of the review and highlights the EOs used and the resulted products with specific biological activity, together with the corresponding reference used.

Polymeric Matrix	EOs	Loading Method/Product Preparation	Activity	Reference
Alginate/soy protein isolate	Thyme	Emulsification/atomization	Antimicrobial/antioxidant	[55]
Alginate/cashew gum	Lippia sidoides	Emulsification	Antimicrobial	[64]
Alginate	Lavender	Emulsification/Electrospinning	Antibacterial/anti-inflammatory	[72]
Alginate/soy or hemp proteins	Green tea extract	Microbeads	Antioxidant	[73]
Sodium alginate/poly(vinyl alcohol)	Cinnamon, clove, lavender	Emulsification/Electrospinning	Antibacterial	[81]
Calcium, aluminum alginate/pectinate	Mentha piperita	Fusion coating procedure	Irritable bowel syndrome treatment	[82]
Soy protein/montmorillonite clays	Eugenol	Suspension/film-forming	Antimicrobial	[56]
Cellulose acetate	Lemongrass	Solvent/non-solvent	Antimicrobial	[85]
Cellulose acetate	Thymol	High pressure techniques	Antibacterial	[86]
Cellulose acetate	Cinnamon/Lemongrass/Peppermint	Electrospinning	Antibacterial	[89]
Cellulose acetate	Rosemary/Oregano	Electrospinning	Antimicrobial	[90]
Ethyl cellulose	Babchi essential oil	Quasi emulsion solvent evaporation	Antimicrobial/antibacterial	[87]
Silk fibroin/Poly (capro-lactone)/Hyaluronic acid	Thymol	Electrospinning	Antimicrobial	[136]
Chitosan	Thymol	Hydrogel	Antimicrobial/antioxidant	[100]
Chitosan	Thyme	Nanoparticles/nanocapsules	Antibacterial	[103]
Chitosan	Rosmarinic acid	Nanoemulsions	Neuroprotective therapy	[104]
Chitosan modified/β-cyclo-dextrin	Cinnamomum zeylanicum (CEO)	Ionic gelation	Ocular mucosa, cornea or transdermal delivery of CEO	[105]
Chitosan/Poly (vinyl alcohol)	Cinnamon leaf/Clove/Eugenol	Films via solvent casting/phase inversion	Antimicrobial	[106]
Chitosan/pentasodium tripolyphosphate/sodium hexametaphosphate	Carum copticum	Emulsion/ionic gelation	Antioxidant/antibacterial	[107]
Chitosan	Peppermint/Green tea	Emulsion/ionic gelation	Antioxidant/antibacterial	[108]
Chitosan	Cardamom	Nanoparticles ionic gelation	Antimicrobial	[109]
Chitosan	Carvacrol	Emulsion/ionic gelation	Antimicrobial	[110]
Whey protein	Cinnamon	Emulsion	Antiseptic, antiviral, antifungal, antioxidant	[135]
Whey protein/maltodextrin	Rosemary	Emulsion	Antiseptic/astringent	[121]
Maltodextrin/octenylsuccinic anhydride	Avocado	Microcapsules	Antioxidant/anti-inflammatory	[122]
Gum acacia/maltodextrin	Lavender	Spray drying	Anti-inflammatory/wound-healing	[123]
Arabic gum/maltodextrin/modified starch	Oregano	Spray drying	Antimicrobial	[125]
Chitosan microbeads	Thyme	Microbeads swelling	Antioxidant/antihypertensive	[101]
Chitosan/tripolyphosphate,	Eugenol	Emulsion/ionic gelation crosslinking	Antioxidant	[113]
Chitosan/tripolyphosphate,	Oregano	Emulsion/ionic gelation	Antioxidant	[116]
Chitosan/Poly(ethylene oxide)	Cinnamaldehyde	Electrospinning	Antimicrobial	[118]
Chitosan/Poly(vinyl alcohol)/ gelatin	*Zataria multiflora*	Electrospinning	Antimicrobial	[119]
Gelatin	*Piper aduncum* L. / *Piper*	Nanoparticle solvent	Antimicrobial	[141]

	hispidinervum C.	evaporation		
Gelatin/Bacterial cellulose polyelectrolyte	Curcumin	Film	Antimicrobia /wound healing	[142]
Starch/Octenyl succinic anhydride	Rosemary	Electrosprayed	Antiseptic/astringent	[128]
Starch	Peppermint	Nanoparticles ultrasonication	Antioxidant/antibacterial	[129]
Starch	Almond	Sprayed micro-encapsulation	Antioxidant	[130]
Modified starch	Lavandula	Supercritical impregnation	Antiinflammatory/antioxid ant	[131]
Porous starch	Clove	3D nanonetwork	Antimicrobial/Antibacterial	[132]
Sago starch/guar gum/whey protein	Carvacrol/citral	Composite films	Antimicrobial: prophylaxis of bacterial gastroenteritis	[133]
Poly(ε-caprolactone)/Poly(lactic acid)	Thymol	Electrospinning	Antibacterial	[145]
Poly(ε-caprolactone /poly(vinyl pyrrolidone)	*Tecomella undulata*	Electrospinning	Antibacterial	[147]
Poly(ε-caprolactone)/poly(vinyl alcohol)	Thyme extract	Coaxial electrospun	Antibacterial	[148]
Poly(caprolactone) / hydroxyapatite	Thymol	Supercritical solvent impregnation	Antibacterial	[149]
Poly(lactic acid)	Linalool	Electrospinning/solution blow spinning	Antibacterial	[151]
Poly(lactic acid)	Candeia	Electrospinning	Antibacterial	[152]
Poly(lactic acid)	Tea Tree/Manuka	Electrospinnig	Antibacterial	[153]
Poly(lactic acid)	Carvacrol	Particle/nano-particle	Antimicrobial	[154]
Poly(lactic acid), poly(methyl methacrylate)	Linalool	Microspheres, solvent evaporation	Antibacterial	[156]
Poly(acrylonitrile)	Lavender	Electrospinnig	Antibacterial	[157]
Poly(vinylpyrrolidone)	Cinnamon	Electrospinnig	Antibacterial	[158]
Propylene Glycol Caprylate/nonionic and hydrophilic surfactants	*Piper cubeba*	Nanoemulsifying	Anti-inflammatory/astringe nt/antiseptic	[159]
Tween	*Saccocalyx satureioides* Coss. et Durieu	Nanoemulsion/High-pressure homogenization	Antioxidant/anticancer	[162]
Tween	*Rosmarinus officinalis*	Nanoemulsion	Anti-inflammatory/antialgi c	[164]
Fish-skin gelatin	Carvacrol	Blow spinning solution	Antimicrobial	[179]
Nanostructured lipid carriers	*Rosmarinus officinalis* /Lavandula/Origanum/Thymus	Phase inversion temperature / high-pressure homogenization	Anti-inflammatory/antioxi dant	[169]

References

55. Fernandes Nassar, S.; Dombre, C.; Gastaldi, E.; Touchaleaume, F.; Chalier, P. Soy protein isolate nanocomposite film enriched with eugenol, an antimicrobial agent: Interactions and properties. *J. Appl. Polym. Sci.* 2018, *135*, 45941, doi:10.1002/app.45941.
56. Barboza, J.N.; da Silva Maia Bezerra Filho, C.; Silva, R.O.; Medeiros, J.V.R.; de Sousa, D.P. An Overview on the Anti-inflammatory Potential and Antioxidant Profile of Eugenol. *Oxid. Med. Cell Longev.* **2018**, *2018*, 3957262, doi:10.1155/2018/3957262.
64. Nayak, A.K.; Ansari, M.T.; Sami, F.; Bera, H.; Hasnain, M.S. Chapter 11—Cashew gum in drug delivery applications. In *Natural Polysaccharides in Drug Delivery and Biomedical Applications*; Hasnain, M.S., Nayak, A.K., Eds.; Academic Press: Cambridge, MA, USA, 2019; pp. 263–283.
72. Belščak-Cvitanović, A.; Đorđević, V.; Karlović, S.; Pavlović, V.; Komes, D.; Ježek, D.; Bugarski, B.; Nedović, V. Protein-reinforced and chitosan-pectin coated alginate microparticles for delivery of flavan-3-ol antioxidants and caffeine from green tea extract. *Food Hydrocoll.* **2015**, *51*, 361–374, doi:10.1016/j.foodhyd.2015.05.039.
73. Kayaci, F.; Uyar, T. Encapsulation of vanillin/cyclodextrin inclusion complex in electrospun polyvinyl alcohol (PVA) nanowebs: Prolonged shelf-life and high temperature stability of vanillin. *Food Chem.* **2012**, *133*, 641–649, doi:10.1016/j.foodchem.2012.01.040.
81. Sibanda, W.; Pillay, V.; Danckwerts, M.P.; Viljoen, A.M.; van Vuuren, S.; Khan, R.A. Experimental design for the formulation and optimization of novel cross-linked oilispheres developed for *in vitro* site-specific release of Mentha piperita oil. *AAPS PharmSciTech* **2004**, *5*, E18, doi:10.1208/pt050118.
82. Stringaro, A.; Colone, M.; Angiolella, L. Antioxidant, Antifungal, Antibiofilm, and Cytotoxic Activities of Mentha spp. Essential Oils. *Medicines* **2018**, *5*, 112, doi:10.3390/medicines5040112.

85. Liakos, I.L.; Iordache, F.; Carzino, R.; Scarpellini, A.; Oneto, M.; Bianchini, P.; Grumezescu, A.M.; Holban, A.M. Cellulose acetate—essential oil nanocapsules with antimicrobial activity for biomedical applications. *Colloids Surf. B Biointerfaces* **2018**, *172*, 471–479, doi:10.1016/j.colsurfb.2018.08.069.
87. Wadhwa, G.; Kumar, S.; Mittal, V.; Rao, R. Encapsulation of babchi essential oil into microsponges: Physicochemical properties, cytotoxic evaluation and anti-microbial activity. *J. Food Drug Anal.* **2019**, *27*, 60–70, doi:10.1016/j.jfda.2018.07.006.
89. Liakos, I.; Rizzello, L.; Hajiali, H.; Brunetti, V.; Carzino, R.; Pompa, P.P.; Athanassiou, A.; Mele, E. Fibrous wound dressings encapsulating essential oils as natural antimicrobial agents. *J. Mater. Chem B* **2015**, *3*, 1583–1589, doi:10.1039/C4TB01974A.
90. Liakos, I.L.; Holban, A.M.; Carzino, R.; Lauciello, S.; Grumezescu, A.M. Electrospun Fiber Pads of Cellulose Acetate and Essential Oils with Antimicrobial Activity. *Nanomaterials* **2017**, *7*, 84, doi:10.3390/nano7040084.
100. Alvarez Echazú, M.I.; Olivetti, C.E.; Anesini, C.; Perez, C.J.; Alvarez, G.S.; Desimone, M.F. Development and evaluation of thymol-chitosan hydrogels with antimicrobial-antioxidant activity for oral local delivery. *Mater. Sci. Eng. C Mater. Biol. Appl.* **2017**, *81*, 588–596, doi:10.1016/j.msec.2017.08.059.
101. Mihailovic-Stanojevic, N.; Belscak-Cvitanovic, A.; Grujic-Milanovic, J.; Ivanov, M.; Jovovic, D.; Bugarski, D.; Miloradovic, Z. Antioxidant and antihypertensive activity of extract from Thymus serpyllum L. in experimental hypertension. *Plant. Foods Hum. Nutr.* **2013**, *68*, 235–240, doi:10.1007/s11130-013-0368-7.
103. Sotelo-Boyas, M.; Correa-Pacheco, Z.; Bautista-Banos, S.; Gomez, Y.G.Y. Release study and inhibitory activity of thyme essential oil-loaded chitosan nanoparticles and nanocapsules against foodborne bacteria. *Int. J. Biol. Macromol.* **2017**, *103*, 409–414, doi:10.1016/j.ijbiomac.2017.05.063.
104. Fachel, F.N.S.; Medeiros-Neves, B.; Dal Prá, M.; Schuh, R.S.; Veras, K.S.; Bassani, V.L.; Koester, L.S.; Henriques, A.T.; Braganhol, E.; Teixeira, H.F. Box-Behnken design optimization of mucoadhesive chitosan-coated nanoemulsions for rosmarinic acid nasal delivery—*In vitro* studies. *Carbohydr. Polym.* **2018**, *199*, 572–582, doi:10.1016/j.carbpol.2018.07.054.
105. Matshetshe, K.I.; Parani, S.; Manki, S.M.; Oluwafemi, O.S. Preparation, characterization and *in vitro* release study of beta-cyclodextrin/chitosan nanoparticles loaded Cinnamomum zeylanicum essential oil. *Int. J. Biol. Macromol.* **2018**, *118*, 676–682, doi:10.1016/j.ijbiomac.2018.06.125.
106. Antunes, J.C.; Tavares, T.D.; Teixeira, M.A.; Teixeira, M.O.; Homem, N.C.; Amorim, M.T.P.; Felgueiras, H.P. Eugenol-Containing Essential Oils Loaded onto Chitosan/Polyvinyl Alcohol Blended Films and Their Ability to Eradicate Staphylococcus aureus or Pseudomonas aeruginosa from Infected Microenvironments. *Pharmaceutics* **2021**, *13*, 195, doi:10.3390/pharmaceutics13020195.
107. Esmaeili, A.; Asgari, A. *In vitro* release and biological activities of Carum copticum essential oil (CEO) loaded chitosan nanoparticles. *Int. J. Biol. Macromol.* **2015**, *81*, 283–290, doi:10.1016/j.ijbiomac.2015.08.010.
108. Shetta, A.; Kegere, J.; Mamdouh, W. Comparative study of encapsulated peppermint and green tea essential oils in chitosan nanoparticles: Encapsulation, thermal stability, in-vitro release, antioxidant and antibacterial activities. *Int. J. Biol. Macromol.* **2019**, *126*, 731–742, doi:10.1016/j.ijbiomac.2018.12.161.
109. Jamil, B.; Abbasi, R.; Abbasi, S.; Imran, M.; Khan, S.U.; Ihsan, A.; Javed, S.; Bokhari, H.; Imran, M. Encapsulation of Cardamom Essential Oil in Chitosan Nano-composites: In-vitro Efficacy on Antibiotic-Resistant Bacterial Pathogens and Cytotoxicity Studies. *Front. Microbiol.* **2016**, *7*, 1580, doi:10.3389/fmicb.2016.01580.
110. Keawchaoon, L.; Yoksan, R. Preparation, characterization and *in vitro* release study of carvacrol-loaded chitosan nanoparticles. *Colloids Surf. B Biointerfaces* **2011**, *84*, 163–171, doi:10.1016/j.colsurfb.2010.12.031.
113. Woranuch, S.; Yoksan, R. Eugenol-loaded chitosan nanoparticles: I. Thermal stability improvement of eugenol through encapsulation. *Carbohydr. Polym.* **2013**, *96*, 578–585, doi:10.1016/j.carbpol.2012.08.117.
116. Hosseini, S.F.; Zandi, M.; Rezaei, M.; Farahmandghavi, F. Two-step method for encapsulation of oregano essential oil in chitosan nanoparticles: Preparation, characterization and *in vitro* release study. *Carbohydr. Polym.* **2013**, *95*, 50–56, doi:10.1016/j.carbpol.2013.02.031.
118. Rieger, K.A.; Schiffman, J.D. Electrospinning an essential oil: Cinnamaldehyde enhances the antimicrobial efficacy of chitosan/poly(ethylene oxide) nanofibers. *Carbohydr. Polym.* **2014**, *113*, 561–568, doi:10.1016/j.carbpol.2014.06.075.
119. Ardekani, N.T.; Khorram, M.; Zomorodian, K.; Yazdanpanah, S.; Veisi, H.; Veisi, H. Evaluation of electrospun poly (vinyl alcohol)-based nanofiber mats incorporated with Zataria multiflora essential oil as potential wound dressing. *Int. J. Biol. Macromol.* **2019**, *125*, 743–750, doi:10.1016/j.ijbiomac.2018.12.085.
121. Turasan, H.; Sahin, S.; Sumnu, G. Encapsulation of rosemary essential oil. *LWT* **2015**, *64*, 112–119, doi:10.1016/j.lwt.2015.05.036.
122. Sotelo-Bautista, M.; Bello-Perez, L.A.; Gonzalez-Soto, R.A.; Yañez-Fernandez, J.; Alvarez-Ramirez, J. OSA-maltodextrin as wall material for encapsulation of essential avocado oil by spray drying. *J. Dispers. Sci. Technol.* **2020**, *41*, 235–242, doi:10.1080/01932691.2018.1562939.
123. Burhan, A.M.; Abdel-Hamid, S.M.; Soliman, M.E.; Sammour, O.A. Optimisation of the microencapsulation of lavender oil by spray drying. *J. Microencapsul.* **2019**, *36*, 250–266, doi:10.1080/02652048.2019.1620355.
125. Partheniadis, I.; Vergkizi, S.; Lazari, D.; Reppas, C.; Nikolakakis, I. Formulation, characterization and antimicrobial activity of tablets of essential oil prepared by compression of spray-dried powder. *J. Drug Deliv. Sci. Technol.* **2019**, *50*, 226–236, doi:10.1016/j.jddst.2019.01.031.

128. Biduski, B.; Kringel, D.H.; Colussi, R.; Hackbart, H.; Lim, L.T.; Dias, A.R.G.; Zavareze, E.D.R. Electrosprayed octenyl succinic anhydride starch capsules for rosemary essential oil encapsulation. *Int. J. Biol. Macromol.* **2019**, *132*, 300–307, doi:10.1016/j.ijbiomac.2019.03.203.
129. Liu, C.; Li, M.; Ji, N.; Liu, J.; Xiong, L.; Sun, Q. Morphology and Characteristics of Starch Nanoparticles Self-Assembled via a Rapid Ultrasonication Method for Peppermint Oil Encapsulation. *J. Agric. Food Chem.* **2017**, *65*, 8363–8373, doi:10.1021/acs.jafc.7b02938.
130. Hoyos-Leyva, J.D.; Bello-Perez, L.A.; Agama-Acevedo, J.E.; Alvarez-Ramirez, J.; Jaramillo-Echeverry, L.M. Characterization of spray drying microencapsulation of almond oil into taro starch spherical aggregates. *LWT* **2019**, *101*, 526–533, doi:10.1016/j.lwt.2018.11.079.
131. Varona, S.; Rodríguez-Rojo, S.; Martín, Á.; Cocero, M.J.; Duarte, C.M.M. Supercritical impregnation of lavandin (Lavandula hybrida) essential oil in modified starch. *J. Supercrit. Fluids* **2011**, *58*, 313–319, doi:10.1016/j.supflu.2011.06.003.
132. Fang, Y.; Fu, J.; Liu, P.; Cu, B. Morphology and characteristics of 3D nanonetwork porous starch-based nanomaterial via a simple sacrifice template approach for clove essential oil encapsulation. *Ind. Crops Prod.* **2020**, *143*, 111939, doi:10.1016/j.indcrop.2019.111939.
133. Vilas Dhumal, C.; Pal, K.; Sarkar, P. Synthesis, characterization, and antimicrobial efficacy of composite films from guar gum/sago starch/whey protein isolate loaded with carvacrol, citral and carvacrol-citral mixture. *J. Mater. Sci. Mater. Med.* **2019**, *30*, 117, doi:10.1007/s10856-019-6317-8.
135. Abdel-Wahhab, M.A.; El-Nekeety, A.A.; Hassan, N.S.; Gibriel, A.A.Y.; Abdel-Wahhab, K.G. Encapsulation of cinnamon essential oil in whey protein enhances the protective effect against single or combined sub-chronic toxicity of fumonisin B(1) and/or aflatoxin B(1) in rats. *Environ. Sci. Pollut. Res. Int.* **2018**, *25*, 29144–29161, doi:10.1007/s11356-018-2921-2.
136. Miguel, S.P.; Simões, D.; Moreira, A.F.; Sequeira, R.S.; Correia, I.J. Production and characterization of electrospun silk fibroin based asymmetric membranes for wound dressing applications. *Int. J. Biol. Macromol.* **2019**, *121*, 524–535, doi:10.1016/j.ijbiomac.2018.10.041.
145. Karami, Z.; Rezaeian, I.; Zahedi, P.; Abdollahi, M. Preparation and performance evaluations of electrospun poly(ε-caprolactone), poly(lactic acid), and their hybrid (50/50) nanofibrous mats containing thymol as an herbal drug for effective wound healing. *J. Appl. Polym. Sci.* **2013**, *129*, 756–766, doi:10.1002/app.38683.
147. Suganya, S.; Senthil Ram, T.; Lakshmi, B.S.; Giridev, V.R. Herbal drug incorporated antibacterial nanofibrous mat fabricated by electrospinning: An excellent matrix for wound dressings. *J. Appl. Polym. Sci.* **2011**, *121*, 2893–2899, doi:10.1002/app.33915.
148. Koushki, P.; Bahrami, S.H.; Ranjbar-Mohammadi, M. Coaxial nanofibers from poly(caprolactone)/ poly(vinyl alcohol)/Thyme and their antibacterial properties. *J. Ind. Text.* **2018**, *47*, 834–852, doi:10.1177/1528083716674906.
149. Ivanovic, J.; Knauer, S.; Fanovich, A.; Milovanovic, S.; Stamenic, M.; Jaeger, P.; Zizovic, I.; Eggers, R. Supercritical CO2 sorption kinetics and thymol impregnation of PCL and PCL-HA. *J. Supercrit. Fluids* **2016**, *107*, 486–498, doi:10.1016/j.supflu.2015.07.001.
151. Souza, M.A.; Oliveira, J.E.; Medeiros, E.S.; Glenn, G.M.; Mattoso, L.H. Controlled Release of Linalool Using Nanofibrous Membranes of Poly(lactic acid) Obtained by Electrospinning and Solution Blow Spinning: A Comparative Study. *J. Nanosci. Nanotechnol.* **2015**, *15*, 5628–5636, doi:10.1166/jnn.2015.9692.
152. Mori, C.L.S.d.O.; dos Passos, N.A.; Oliveira, J.E.; Altoé, T.F.; Mori, F.A.; Mattoso, L.H.C.; Scolforo, J.R.; Tonoli, G.H.D. Nanostructured Polylactic Acid/Candeia Essential Oil Mats Obtained by Electrospinning. *J. Nanomater.* **2015**, *2015*, 439253, doi:10.1155/2015/439253.
153. Zhang, W.; Huang, C.; Kusmartseva, O.; Thomas, N.L.; Mele, E. Electrospinning of polylactic acid fibres containing tea tree and manuka oil. *React. Funct. Polym.* **2017**, *117*, 106–111, doi:10.1016/j.reactfunctpolym.2017.06.013.
154. Scaffaro, R.; Lopresti, F.; Marino, A.; Nostro, A. Antimicrobial additives for poly(lactic acid) materials and their applications: Current state and perspectives. *Appl. Microbiol. Biotechnol.* **2018**, *102*, 7739–7756, doi:10.1007/s00253-018-9220-1.
155. Scaffaro, R.; Maio, A.; Nostro, A. Poly(lactic acid)/carvacrol-based materials: Preparation, physicochemical properties, and antimicrobial activity. *Appl. Microbiol. Biotechnol.* **2020**, *104*, 1823–1835, doi:10.1007/s00253-019-10337-9.
156. Dusankova, M.; Pummerova, M.; Sedlarik, V. Microspheres of essential oil in polylactic acid and poly(methyl methacrylate) matrices and their blends. *J. Microencapsul.* **2019**, *36*, 305–316, doi:10.1080/02652048.2019.1623337.
157. Balasubramanian, K.; Kodam, K.M. Encapsulation of therapeutic lavender oil in an electrolyte assisted polyacrylonitrile nanofibres for antibacterial applications. *RSC Adv.* **2014**, *4*, 54892–54901, doi:10.1039/C4RA09425E.
158. Kesici Güler, H.; Cengiz Çallıoğlu, F.; Sesli Çetin, E. Antibacterial PVP/cinnamon essential oil nanofibers by emulsion electrospinning. *J. Text. Inst.* **2019**, *110*, 302–310, doi:10.1080/00405000.2018.1477237.
159. Shakeel, F.; Shazly, G.A.; Raish, M.; Ahmad, A.; Kalam, M.A.; Ali, N.; Ansari, M.A.; Elosaily, G.M. Biological investigation of a supersaturated self-nanoemulsifying drug delivery system of Piper cubeba essential oil. *RSC Adv.* **2015**, *5*, 105206–105217, doi:10.1039/C5RA22900F.
162. Aouf, A.; Ali, H.; Al-Khalifa, A.R.; Mahmoud, K.F.; Farouk, A. Influence of Nanoencapsulation Using High-Pressure Homogenization on the Volatile Constituents and Anticancer and Antioxidant Activities of Algerian Saccocalyx satureioides Coss. et Durieu. *Molecules* **2020**, *25*, 756, doi:10.3390/molecules25204756.

164. Borges, R.S.; Lima, E.S.; Keita, H.; Ferreira, I.M.; Fernandes, C.P.; Cruz, R.A.S.; Duarte, J.L.; Velázquez-Moyado, J.; Ortiz, B.L.S.; Castro, A.N.; et al. Anti-inflammatory and antialgic actions of a nanoemulsion of Rosmarinus officinalis L. essential oil and a molecular docking study of its major chemical constituents. *Inflammopharmacology* **2018**, *26*, 183–195, doi:10.1007/s10787-017-0374-8.
169. Carbone, C.; Martins-Gomes, C.; Caddeo, C.; Silva, A.M.; Musumeci, T.; Pignatello, R.; Puglisi, G.; Souto, E.B. Mediterranean essential oils as precious matrix components and active ingredients of lipid nanoparticles. *Int. J. Pharm.* **2018**, *548*, 217–226, doi:10.1016/j.ijpharm.2018.06.064.
179. El-Attar, N.E.; Hassan, M.K.; Alghamdi, O.A.; Awad, W.A. Deep learning model for classification and bioactivity prediction of essential oil-producing plants from Egypt. *Sci. Rep.* **2020**, *10*, 21349, doi:10.1038/s41598-020-78449-1.
